# Prioritising Causal Genes at Type 2 Diabetes Risk Loci

**DOI:** 10.1007/s11892-017-0907-y

**Published:** 2017-07-31

**Authors:** Antje K. Grotz, Anna L. Gloyn, Soren K. Thomsen

**Affiliations:** 1 Oxford Centre for Diabetes, Endocrinology & Metabolism, University of Oxford, Oxford, UK; 2 Wellcome Trust Centre for Human Genetics, University of Oxford, Oxford, UK; 3 National Institute of Health Research Oxford Biomedical Research Centre, Churchill Hospital, Oxford, UK

**Keywords:** Genome-wide association study, Type 2 diabetes, Genetic mechanism, Functional genomics, Causal gene, Effector transcript

## Abstract

**Purpose of Review:**

Genome-wide association studies (GWAS) for type 2 diabetes (T2D) risk have identified a large number of genetic loci associated with disease susceptibility. However, progress moving from association signals through causal genes to functional understanding has so far been slow, hindering clinical translation. This review discusses the benefits and limitations of emerging, unbiased approaches for prioritising causal genes at T2D risk loci.

**Recent Findings:**

Candidate causal genes can be identified by a number of different strategies that rely on genetic data, genomic annotations, and functional screening of selected genes. To overcome the limitations of each particular method, integration of multiple data sets is proving essential for establishing confidence in the prioritised genes. Previous studies have also highlighted the need to support these efforts through identification of causal variants and disease-relevant tissues.

**Summary:**

Prioritisation of causal genes at T2D risk loci by integrating complementary lines of evidence promises to accelerate our understanding of disease pathology and promote translation into new therapeutics.

## Introduction

In the last decade, genome-wide association studies (GWAS) have evolved as a powerful tool for deciphering the genetic component of type 2 diabetes (T2D) risk. By associating regions of the genome with disease susceptibility, more than 100 loci influencing T2D risk have been identified so far [1–6, 7••]. Moving on from an era of disease locus discovery, post-GWAS methodologies are now advancing to functionally characterise the underlying genes and to interrogate disease pathways. These comprehensive efforts promise to enable subsequent translation into improved disease diagnostics, treatment, and prevention. However, the progression from association signals at T2D loci to causal genes and a functional understanding of diabetes pathology has been limited. The slow progress is due, in part, to problems arising from the methodology itself and, in part, a consequence of the underlying nature of the association signals.

GWAS exploit the fact that single-nucleotide polymorphisms (SNP) tend to be located in linkage disequilibrium (LD) with other variants [8]. By analysing SNPs that lie in LD with non-genotyped variants, these can serve as representatives for their haplotype (‘tag SNPs’), and it is thus possible to achieve reasonable genome-wide coverage of common variation by analysing between 0.5–1 million SNPs [9–12]. Thus, the GWAS paradigm is designed to detect SNPs that act as a proxy for disease-associated regions or loci, and not necessarily the actual causal variants. Additionally, the majority of association signals (~90%) are found in non-coding regions, presumably influencing disease risk through effects on gene regulation [13]. The detected SNPs in non-coding regions are named after the nearest protein-coding gene, but proximity to a gene does not imply causality.

The challenge for functional follow-up studies in elucidating disease mechanisms lies therefore in finding both causal variants and the genes through which they impact on disease risk for the corresponding SNPs. Here, we first discuss the benefits of determining the causal variant(s) and affected tissue(s) as a prerequisite for identifying effector transcripts. We review several approaches for prioritising causal genes at T2D loci and provide recent and prominent examples of likely effector transcripts identified by these strategies. Finally, we highlight the importance of triangulating from multiple datasets and discuss the prospects for future integrative studies.

## Prerequisites for Finding Causal Genes

Uncovering the underlying causal mechanisms of T2D risk loci is not exclusively a matter of finding causal genes, since these efforts are complicated by the need to identify both causal variant(s) and the affected tissue(s) in order to obtain a complete picture of disease pathology. Moreover, this additional information is often an inevitable requirement for performing functional follow-up studies in an appropriate model system.

### Causal Variants

In GWAS, the variant most strongly associated with disease risk is reported for each locus, though such ‘lead SNPs’ may only serve as surrogate markers for other genetic perturbations that directly contribute to disease pathology. Identifying the true causal variants can provide a direct functional link between genotype and the observed disease phenotype, especially in cases where the variant is protein altering. To identify a causal variant, or a set of likely causal variants, several strategies have been developed, including fine-mapping of disease-associated regions, experimental prioritisation, and in silico prediction tools.

Fine-mapping of a locus involves analysing SNPs in a defined region of the genome for disease association and is used to refine a GWAS association signal from the surrogate lead SNP to the actual causal variant(s). The SNPs are assayed by deep sequencing, or custom array-genotyping based on GWAS variants and imputation from extensive sequencing efforts such as the 1000 Genomes Project [14, 15]. To achieve sufficient statistical power to detect the association of the true causal variant, large sample sizes are required and the studies often include populations drawn from diverse ancestries to exploit differences in LD patterns [16].

Even so, most fine-mapping efforts uncover a large number of variants that, between them, are likely to be driving a particular association signal—a so-called credible set. In some exceptional cases, however, it is possible to narrow down the credible set to a single variant, as is the case for the melatonin receptor 1B gene (*MTNR1B*) [17•]. The *MTNR1B* locus has previously been implicated in T2D risk and the identification of the single causal variant revealed a likely, direct functional link to the causal gene [18]. The risk allele creates a binding site for the transcription factor NEUROD1 and is associated with preferential binding in human pancreatic beta cells. This additional transcription factor binding event also implicates increased FOXA2-bound enhancer activity and *MTNR1B* expression.

Another way to approach the search for causal variants at GWAS loci is by experimentally testing prioritised SNPs. This strategy was, for example, pursued at the *JAZF1* and *CDC123*/*CAMK1D* loci [19–21]. Variants in high LD (*r* ^2^ > 0.8) with the lead GWAS SNP were selected for functional analysis based on maps of open chromatin. Effects on gene expression were tested in luciferase reporter assays, and DNA binding capability was analysed through electrophoretic mobility shift assays. The identified potential causal variants at the *JAZF1* and *CDC123*/*CAMK1D* loci appear to act as part of cis-regulatory modules (CRMs). These specific regions harbour combinatorial transcription factor binding sites (TFBS), and the variants affect binding of PDX1 and FOXA1/FOXA2, respectively. However, due to practical limitations, this type of experimental studies mostly analyses a subset of regional variants, opening up the possibility of missing potential true causal variants. Further, the evidence generated is only circumstantial, since establishing functionality is necessary but not sufficient to prove causation. The emergence of new experimental lines of evidence may affect the prioritisation of the true causal variants and should ideally involve integration of different types of analyses (see section on “Integrative approach”).

To overcome the practical limitations of functional approaches for identifying causal variants, in silico prediction tools offer an alternative method based on specific assumptions regarding their properties. A recent study, for example, leveraged phylogenetic conservation of TFBS within CRMs to predict causal variants at the *PPARG* and *FTO* T2D risk loci [22, 23•]. This computational approach, termed phylogenetic module complexity analysis (PMCA), identified a clustering of homeobox TFBS at T2D risk loci, and initially proposed a potential causal variant at the *PPARG* locus, which allowed for a subsequent functional interpretation [22]. The risk allele at *PPARG2* leads to enhanced binding of the repressive homeobox transcription factor PRRX1, and thus reduced *PPARG2* expression, defective lipid handling, and insulin sensitivity. PMCA was also successfully applied to identify the causal variant and a potential disease mechanism at the obesity-associated *FTO* locus, a region showing the strongest genetic association in GWAS for obesity and body mass index traits [24, 25]. The proposed causal allele was shown to alter an ARID5B repressor motif, leading to activation of the distant *IRX3* and *IRX5* in adipocyte precursor cells, and pro-obesity consequences for adipocyte thermogenesis regulation [23•]. This work also highlights the additional complexity arising from having multiple causal genes for disease-associated haplotypes. Though post-GWAS efforts have tended to focus on the idea of a single causal gene per locus, causal variant(s) may influence any number of regional genes, and not necessarily in the same manner across different contexts.

### Causal Contexts

An important aspect of the prioritisation of causal genes and variants at GWAS loci is to consider the appropriate tissue(s) and developmental stage(s), which allow any functional follow-up studies to be performed in a disease-relevant model. As the majority of T2D association signals are located in non-coding regions and exert regulatory effects, their influence on gene expression may be subject to context-specific activity [26]. Thus, studies analysing the implicated variants and genes need to consider the surrounding genomic context and expression patterns. A notable example is provided by work on the *PTF1A* gene, where a disease-relevant model, human pancreatic progenitor cells, was critical to elucidating a mechanism for isolated pancreatic agenesis [27•]. The identified mutations were found to disrupt an enhancer region that is selectively active in pancreatic progenitor cells and, importantly, show no activity in corresponding adult cell lines.

## Strategies for Prioritising Causal Genes

The aim of translating genetic variants into molecular mechanisms will ultimately centre on the identification of causal genes. It is enhanced understanding at this level that holds the key to discovering novel treatments, prevention targets, and diagnostic markers. Several strategies to address this issue are being pursued, including the interrogation of coding variants, establishing variant-gene links for non-coding variants, and using high-throughput screens to prioritise candidate genes.

### Coding Variants

**Fig. 1:**
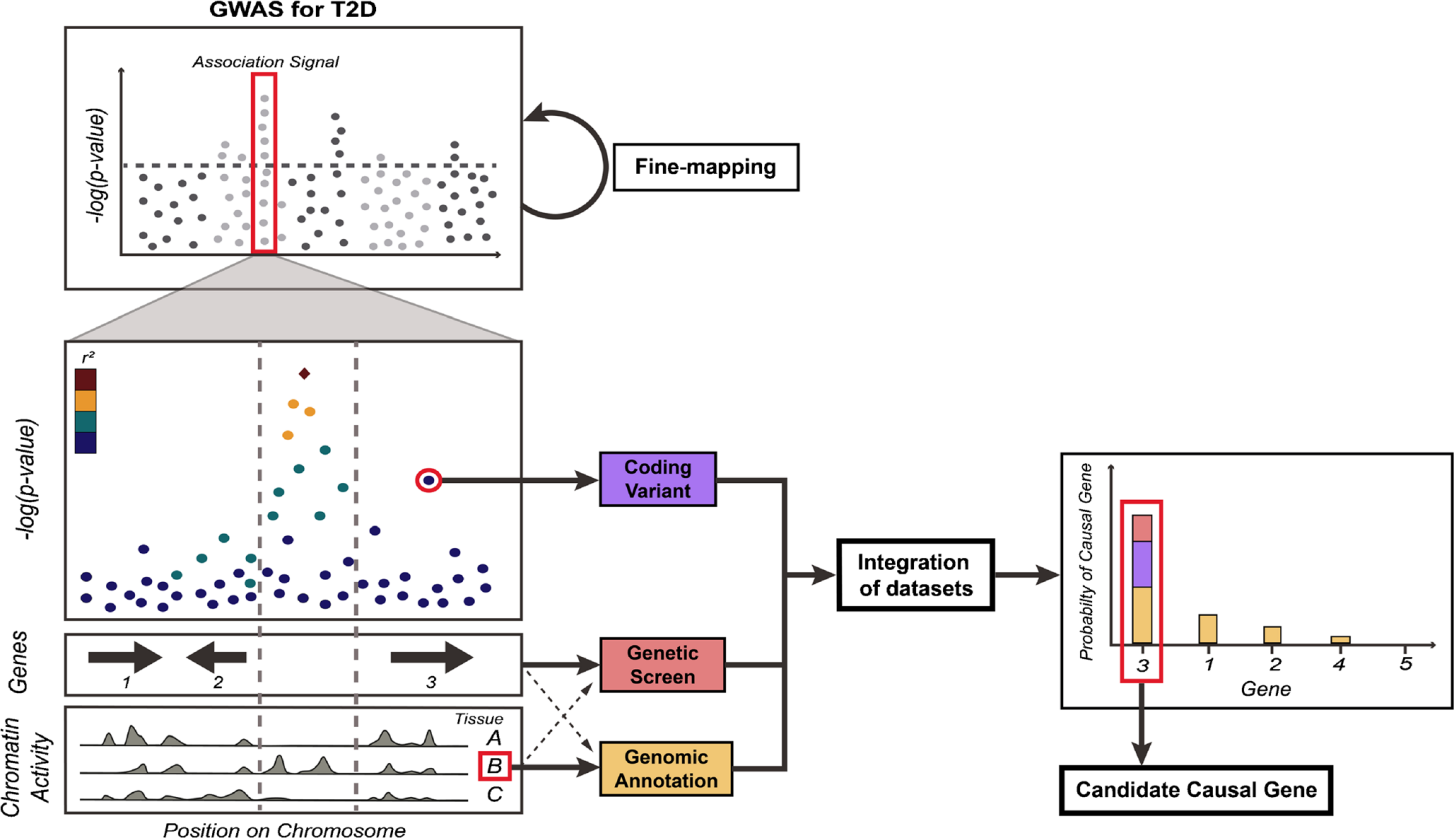
Using genetic data, genomic annotations, and functional screening for prioritising causal genes at T2D GWAS loci. GWAS for T2D risk have identified more than 100 independent association signals to date (Manhattan plot; *top left*), but the majority of causal genes driving the effects on disease susceptibility remain unknown. Fine-mapping of associated regions can aid the prioritisation efforts by narrowing down the credible sets of causal variants (see main text). Emerging strategies for prioritising causal genes are highlighted for a hypothetical T2D risk locus (*bottom left*); the regional association plot shows a primary, non-coding association signal located upstream of gene 2 and downstream of gene 3 (lead variant; *red diamond*). An independent, coding variant in gene 3 displays moderate (sub-significant) association with T2D risk, providing evidence hinting at this gene as causal at this locus. Further, genomic annotations for different cell types (A, B, and C, for illustration) reveal the primary association signal to be located in a region that displays tissue-specific activity in cell type B. This information provides valuable information for two independent prioritisation strategies. Firstly, functional genetic screening of all regional genes (e.g. genes 1–3 [shown] and 4–5 [not shown]) can be performed in a disease-relevant context, measuring a phenotype specific to cell type B. Further, variant-gene links can be established through experimental studies in tissue B, using, for example, cis-eQTL or chromatin confirmation capture methodologies. Importantly, each of the methods outlined have their own set of limitations (see main text), and integration is thus important for establishing confidence in particular candidates. In this case (graph; *bottom right*), gene 3, which was highlighted by genetic data (*purple bar*), has also been found in a functional screen to cause defects in a disease-relevant tissue, adding further evidence in support of this gene as causal (*red bar*). Finally, variant-gene annotations have shown some degree of evidence for associations between the non-coding signal and genes 1–4 (*yellow bars*), with gene 3 being the most significant target. Taken together, the aggregate burden of priors provides a high degree of confidence in gene 3 as the candidate causal gene at this locus, which can be used to prioritise the gene for follow-up in-depth validation studies

Recent GWAS endeavours have shifted attention towards exome-arrays and exome-sequencing to enable identification of rare and low-frequency variants with potentially larger effect sizes—and a more direct biological interpretation—than common variants [7••, 28–30]. Missense variants in coding regions have a protein-altering effect that can directly pinpoint causal genes, offering the possibility of a straightforward and rapid translation into the clinic (Fig. 1).

The importance of coding variants for ascertaining causal mechanisms is illustrated by *SLC30A8*, which encodes a zinc transporter (ZnT8) that is active in the secretory vesicles of beta cells. *SLC30A8* was initially identified as a T2D susceptibility gene harbouring a common missense variant [2]. Contradictory to the supposed negative impact of this risk allele, recent efforts to identify protein-truncating variants leading to loss of function (LOF) in T2D genes discovered several rare protein-truncating variants in *SLC30A8* [31•]. Strikingly, the haploinsufficiency conferred by this class of variants was found to be associated with a 65% reduction in T2D risk. By discovering multiple independent coding variants at this GWAS locus, *SLC30A8* has been validated with high confidence as the causal gene. Furthermore, this study highlights the importance of discovering an extended allelic series to understand functional mechanisms. More broadly, it has established reduced activity of ZnT8 as a protective disease mechanism in T2D and a potential treatment strategy based on antagonism [32].

The power to detect causal genes through coding variants can be further harnessed by performing genetic association studies in isolated populations. These populations, founded by a bottleneck event, show a higher degree of LD, less genetic complexity, and higher allelic frequencies due to genetic drift, which leads to fixation or extinction of specific alleles over time [33]. Furthermore, these studies also benefit from shared non-genetic backgrounds (e.g. common lifestyle and cultural habits), which is a potential confounding factor in larger outbred populations [34]. Exploiting these advantages of studies in isolated populations, a nonsense coding variant in *TBC1D4* was discovered in the Greenlandic population with the largest effect size for a common T2D risk allele (odds ratio = 10.3) [35•]. The variant disrupts the full-length isoform of TBC1D4, which is selectively expressed in skeletal muscle, thus exerting its influence on T2D risk through insulin resistance.

Another recent study leveraging the advantages of isolated populations detected a low-frequency coding variant in *AKT2* in the Finnish population [36]. The allele confers T2D risk through increased fasting plasma insulin levels and expands the allelic spectrum from the previously known rare variants in *AKT2* that cause monogenic heterogeneous glycaemic diseases [37, 38]. Collectively, these studies illustrate the importance of identifying coding variants—in isolated and outbred populations—for straightforward translation into molecular mechanisms. While harnessing coding variation can offer powerful insights into causal mechanism, this approach is fundamentally limited by the occurrence of natural variation (in outbred and isolated populations) which necessitates ever-larger association studies to detect rare, coding variation. In addition, identification of a coding signal is not a guarantee for causality, and conditional analysis is often required to estimate the likelihood of a given variant being causal [39]. By design, exome-based studies analyse coding regions only, and thus require additional fine-mapping of non-coding regions to exclude the contribution of non-coding variants as drivers of the association signal.

### Establishing Variant-Gene Links

In contrast to missense coding variants, associating GWAS signals in non-coding regions with their downstream causal gene is often a more complex challenge. To identify regulatory effects, non-coding variants can be correlated with genomic annotations to establish a functional link with their target gene (Fig. 1). Expression quantitative trait loci (eQTLs), for example, describe variants that influence gene expression in close proximity (*cis*-eQTL) or over a long distance (*trans*-eQTL), and provide an approach for directly linking a GWAS variant to its causal gene through effects on expression levels [40]. Crucial for the success of eQTL studies is the interrogation of the correct disease-relevant context(s), since gene expression is often regulated in a cell-type specific manner [41].

For T2D, a large number of disease risk loci have been found through physiological studies to affect insulin processing or secretion in the beta cell, highlighting pancreatic islets as a relevant starting point for annotation studies at these loci [42]. Up to now, islet sample availability has been limiting for large-scale studies, thereby reducing statistical power to detect associations. Nonetheless, recent studies succeeded in mapping islet *cis*-eQTLs and overlapping these with variants driving T2D association signals [43, 44•]. One such coincident locus is *ZMIZ1*, harbouring a gene that had been sparsely characterised for its role in T2D risk [44•]. A recent study confirmed *ZMIZ1* as the likely causal gene at this T2D risk locus, and functional follow-up work has established a role in beta cell function for insulin secretion and exocytosis, thus giving first insights into a potential mechanism [44•, 45].

Tissue availability has so far prevented any progress in finding islet *trans*-eQTLs. T*rans*-eQTLs act over distance and the entire genome is interrogated for any variant-gene associations, thus further limiting power due to more stringent multiple-testing correction [40]. Still, efforts in adipose tissue have demonstrated the power of this approach by elucidating a trans-regulatory network of *KFL14*, a gene linked with both T2D and other metabolic traits [46]. As KLF14 is a transcription factor, the aim of the study was to identify *trans-*genes that are influenced by varied KLF14 levels through *cis*-eQTL variants. Several genes with genome-wide significance were discovered and the study not only connected GWAS, *cis*- and *trans*-associations for the same set of variants, but also defined important disease-related pathways.

The search for causal genes has been pushed ahead by eQTL studies, but the ability to perform large-scale studies containing correlated sets of genotype, phenotype and expression data are still limited by cost obstacles and sample availability. GWAS only measure genetic variation related to a disease phenotype, and expression studies suffer from reduced statistical power due to smaller sample sizes. Predicted expression association studies attempt to circumvent these limitations by integrating existing GWAS and eQTL data [47–50]. This approach aims to identify disease associations based on groups of variants that influence gene expression, directly pinpointing the causal gene instead of tag SNPs. To combine limited available expression sets with large-scale GWAS data, these studies rely on predicted expression modelled from reference panels. The models then impute expression either for publically available summary GWAS data (most large-scale studies) or GWAS data with individual genotypes [47, 49]. This drastically increases power to detect genes that are predicted to show differences in genotype-dependent expression patterns in T2D, and reduces potential confounding factors like reverse causation, where the phenotype and environment influence gene expression [50]. However, similar to *cis*-eQTL studies, predicted expression association studies are unable to detect context-dependent effects that are not captured by the tissues and developmental stages included in the reference panel used for modelling [48, 49]. It is also not possible to exclude the possibility of pleiotropy caused by multiple, correlated effects of groups of variants on gene expression [48]. Despite such limitations, these methods offer a complementary and powerful approach for prioritisation of causal genes and predicted directions of effect.

### Genetic Screening of T2D Genes

A third way to identify genes involved in disease risk is prioritisation based on known or observed functions that are perceived to be relevant for disease pathogenesis. T2D risk variants, for instance, would be expected to affect genes involved in cellular processes relevant to disease susceptibility, such as beta cell function and insulin resistance. A gene found to regulate insulin secretion would thus have high prior odds of being the downstream mediator for a nearby T2D association signal known to impact on islet function. Though this is an indirect approach for prioritisation, the strategy benefits from focusing on the relevant processes that ultimately causes effects on disease pathology (Fig. 1). For unbiased generation of priors, all disease-relevant phenotypes should ideally be comprehensively interrogated in a genome-wide fashion. However, most post-GWAS approaches have previously focused on individual candidate genes, with experimental setups that make them poorly suited for systematic assessment of large numbers of genes across multiple tissues.

High-throughput functional genomic screening is an emerging and increasingly powerful approach that allows for highly parallel phenotypic screening to address this gap. Several screening strategies have been established that differ in their direction of modulated gene expression (gain of function (GOF) vs LOF), format (pooled vs arrayed), and gene modulation techniques (RNA interference (RNAi) vs CRISPR/Cas9 modulation) [51–56, 57•]. Screens can either be performed genome-wide, representing an unbiased approach to detect genes that are involved in a specific phenotype, or based on selected genes of interest. A recent study by Thomsen et al. successfully pursued a small interfering RNA (siRNA) arrayed screening approach to systematically interrogate positional candidate genes at T2D GWAS loci in a human beta cell line [45]. Genes located within 1 Mb of 75 GWAS association signals were analysed for insulin secretion and cell proliferation to reflect beta cell dysfunction. This strategic approach provided 300 genes for screening and identified 45 genes at 37 GWAS loci for having a role in beta cell dysfunction, thus also pinpointing them as potential effector transcripts at these disease loci. Several prioritised genes with poorly characterised connection to beta cell function were separately validated in functional follow-up work including *ARL15*, *THADA*, and *ZMIZ1*. Independently of the previously described *cis*-eQTL study, this work thus attributed a role to *ZMIZ1* in beta cell function, converging multiple lines of evidence to enhance confidence in the candidacy of this gene as causal. Importantly, the study also demonstrated a strong enrichment for known regulators of insulin secretion among significant hits, providing an internal validation that is an essential aspect of any screening strategy.

Taking a more inclusive approach, Pappalardo and colleagues recently pursued the first whole-genome siRNA screen to identify genes involved in glucose homeostasis and T2D [58]. While allowing for a more unbiased approach, performing an arrayed, genome-wide screen restricts the complexity of the phenotype(s) that can be practically measured. This screen focused on a reporter gene readout for insulin promoter activity in a rat beta cell line. The authors were able to identify several novel regulators of insulin promoter activity including *Spry2*, the gene in the closest proximity to a nearby T2D GWAS association signal [59]. The work thus highlights *Spry2* as the likely causal gene at this locus, and follow-up work in cellular and in vivo systems including beta cell specific knockout mice discovered a potential functional mechanism. However, a link between the non-coding association signal and *Spry2* remains to be investigated, ideally through integration with variant-to-gene approaches in human beta cells. This screen also provided robust internal validation by confirming the strongest hits to be known transcription factors targeting the insulin promoter.

Medium-throughput screens and systematic analysis of selected classes of genes represents a related strategy for analysing candidate genes in more depth across a larger spectrum of possible disease phenotypes. This approach was pursued by a recent study that investigated the function of 12 long non-coding RNAs (lncRNAs) in beta cell gene regulation and their potential role in T2D [60]. These lncRNA knockdown targets were selected based on criteria that included expression in a relevant model and an active chromatin profile. The study showed that the beta cell specific lncRNAs jointly regulate enhancer-cluster associated genes with known transcription factors. The lncRNA named as *PLUTO* was established as a regulator of its neighbouring gene *PDX1*, a transcription factor involved in pancreatic development and beta cell function [61]. Based on this overlapping role of lncRNAs and islet transcription factors, and the well-established involvement of the latter in T2D, the work hints at a similarly important role of lncRNAs in T2D pathology.

Future genetic screens hold the potential to play an important role in identifying causal genes for T2D. Pooled approaches are able to extend the scale of arrayed screens in a cost-effective manner and allow for simultaneous perturbation of thousands of genes to promote unbiased interrogation of candidate causal genes. The continuous development and improvement of the differentiation process of induced pluripotent stem cells into beta cells will also allow for investigations of disease-relevant phenotypes at various developmental stages [62, 63]. High-throughput screens thus offer the opportunity to facilitate the transition from T2D GWAS association signals to individual functional follow-up studies by prioritising candidate causal genes based on functional data.

## Integrative Approach

All of the above outlined strategies provide complementary approaches for prioritising causal genes for association signals, each with individual advantages and drawbacks. Coding variants are reliant upon large-scale association studies and naturally occurring variation, while variant-gene links are limited by the availability of primary tissue and possible pleiotropy, and gene-centric functional studies establish indirect evidence in a manner that is strongly dependent on context-dependent effects. As a result, one specific line of evidence can only give limited insights into causal mechanisms and is rarely sufficient to provide definitive evidence for a particular mechanism. The true causal gene(s) can only be identified with confidence through integration and convergence of several complementing datasets [64].

The importance of taking an integrative approach is illustrated by the T2D susceptibility locus on chromosome 11q13, which is located near the protein-coding genes *ARAP1*(*CENTD2*) and *STARD10* [3, 65]. Initial studies highlighted *ARAP1* as an effector transcript at the locus, but recent findings contradict this assumption and instead propose *STARD10* as the causal gene [44, 66, 67••]. Fine-mapping, functional annotation data, chromatin accessibility and conformation capture data, promoter-reporter assays in beta cell models, *cis*-eQTL in islet samples, and global and selective mice knockout models were all used to generate complementary data that attribute a role to *STARD10* at this locus. The comprehensive set of data makes it possible to infer causality by triangulation from different results. This point is emphasised by examining the chromatin confirmation capture data in isolation. Physical interactions between both the *STARD10* and *ARAP1* promoters and variants in the credible causal set highlight the possibility of regulatory effects on either gene. Thus, additional information was required to clarify the roles of these genes in disease pathology.

Another recent study outlines how the integration of genomic, expression and functional data can prioritise a potential causal gene and disease mechanism, and furthermore directly propose a therapeutic hypothesis. The investigated T2D risk allele is common in Mexicans and Latin Americans (~30% allelic frequency) and located near *SLC16A11* and *SLC16A13* [68]. Fine-mapping identified a credible set of causal variants including non-coding variants and missense coding variants in *SLC16A11*. Liver expression data and chromatin modification analysis showed reduced *SLC16A11* expression and less-activating histone modifications in samples from T2D risk allele carriers, thus proposing *SLC16A11* as the candidate causal gene. Further studies into the function of SLC16A11, an H+-coupled monocarboxylate transporter revealed that the coding risk variants exert their effect through decreased chaperone interaction and SLC16A11 plasma membrane localization. Rusu et al. were also able to show how decreased SLC16A11 function might lead to increased T2D risk by having an effect on cellular fatty acid and lipid metabolism, providing a possible therapeutic strategy.

Despite comprehensive integration of datasets, the evidence in these studies still cannot exclude additional pleiotropy (e.g. regulatory effects that remain undetected due to insufficient power, or effects that manifest in cell types not studied). Exhaustively addressing these gaps will require access to data that enable interrogation of variant function in any context (e.g. well-powered cis-eQTL studies across all disease-relevant cell states), and is far from being a feasible aim for current post-GWAS studies. The emergence of ever-greater, publically available datasets of this nature will increasingly facilitate integration with results of individual studies and thereby guide interpretation. Large-scale projects such as ENCODE, Genotype-Tissue Expression (GTEx) and the NIH Epigenomics Roadmap have already generated enormous functional annotation datasets that allow for intersection with potentially causal variants across hundreds of cell types [41, 69, 70]. However, in the case of tissue-specific annotations for inaccessible tissues like islets, these datasets are often lacking or immature. Future studies will expand the possibilities of integrating datasets and improve the prospects for identification of causal genes in T2D.

## Conclusion

Connecting GWAS association signals to their corresponding causal genes has proven a major experimental challenge and bottleneck for therapeutic translation. As a consequence of GWAS design and the genetic architecture of T2D, causal variants and genes cannot be easily inferred from genetic association studies, hindering functional interpretation. Thus, prioritising causal genes at T2D loci to aid functional understanding is a central aspect of current studies. These studies must be guided by parallel efforts to identify causal variants and appropriate disease-relevant model systems. A number of strategies have emerged for causal gene prioritisation based on genetic data, genomic annotations, and functional screening, each with limitations that render them insufficient in isolation. Several lines of evidence and different experimental strategies should thus be triangulated to validate the results and increase confidence in a specific causal mechanism. Looking forward, this era of gene prioritisation based on T2D GWAS loci and functional understanding holds the promise to unlock the full potential of genomic medicine and clinical translation.

## Abbreviations

CRISPR: Clustered Regularly Interspaced Short Palindromic Repeats
CRM: *Cis*-regulatory modules
ENCODE: Encyclopaedia of DNA elements
eQTLs: Expression quantitative trait loci
GOF: Gain of function
GWAS: Genome-wide association studies
LD: Linkage disequilibrium
lncRNAs: Long non-coding RNAs
LOF: Loss of function
MTNR1B: Melatonin receptor 1B
PMCA: Phylogenetic module complexity analysis
RNAi: RNA interference
siRNA: Small interfering RNA
SNP: Single-nucleotide polymorphism
TFBS: Transcription factor binding sites
T2D: Type 2 diabetes
